# Supplementary material for: The Formation of Mn-Ce Oxide Catalysts for CO Oxidation by Oxalate Route: The Role of Manganese Content
Source: Nanomaterials (Basel). 2021 Apr 12;11(4):988. doi: 10.3390/nano11040988 (PMC8070498; doi:10.3390/nano11040988)
Supplement: Supplementary file 1 [file nanomaterials-11-00988-s001.pdf]

Supplementary Information

The formation of nanostructured Mn-Ce oxide catalysts for CO oxidation by oxalate route: role of manganese content

Dissolution of the test samples was carried out by stoichiographic titration [1], in dynamic mode, with a time-programmed change in the composition and concentration of the solvent and temperature, passing from the initial “soft” to the final “hard” conditions. Practically stoichiographic titration consists in regulating the speed of the stoichiograph's peristaltic pumps using an electronic device, which leads to the necessary change in the composition and concentration of solvents directly during the dissolution of the substance. The temperature is controlled using a thermostat. The dissolution conditions are manually controlled and depends on the actual observed process of dissolution.

There are the following rules:

1. If the dissolution process is slow or stops, then the concentration of the solvent and / or temperature is increased, or the composition of the solvent is changed.

2. If the process of dissolution of at least one element proceeds at a significant rate, changes in the concentration and temperature of the solvent reduce or completely stop.

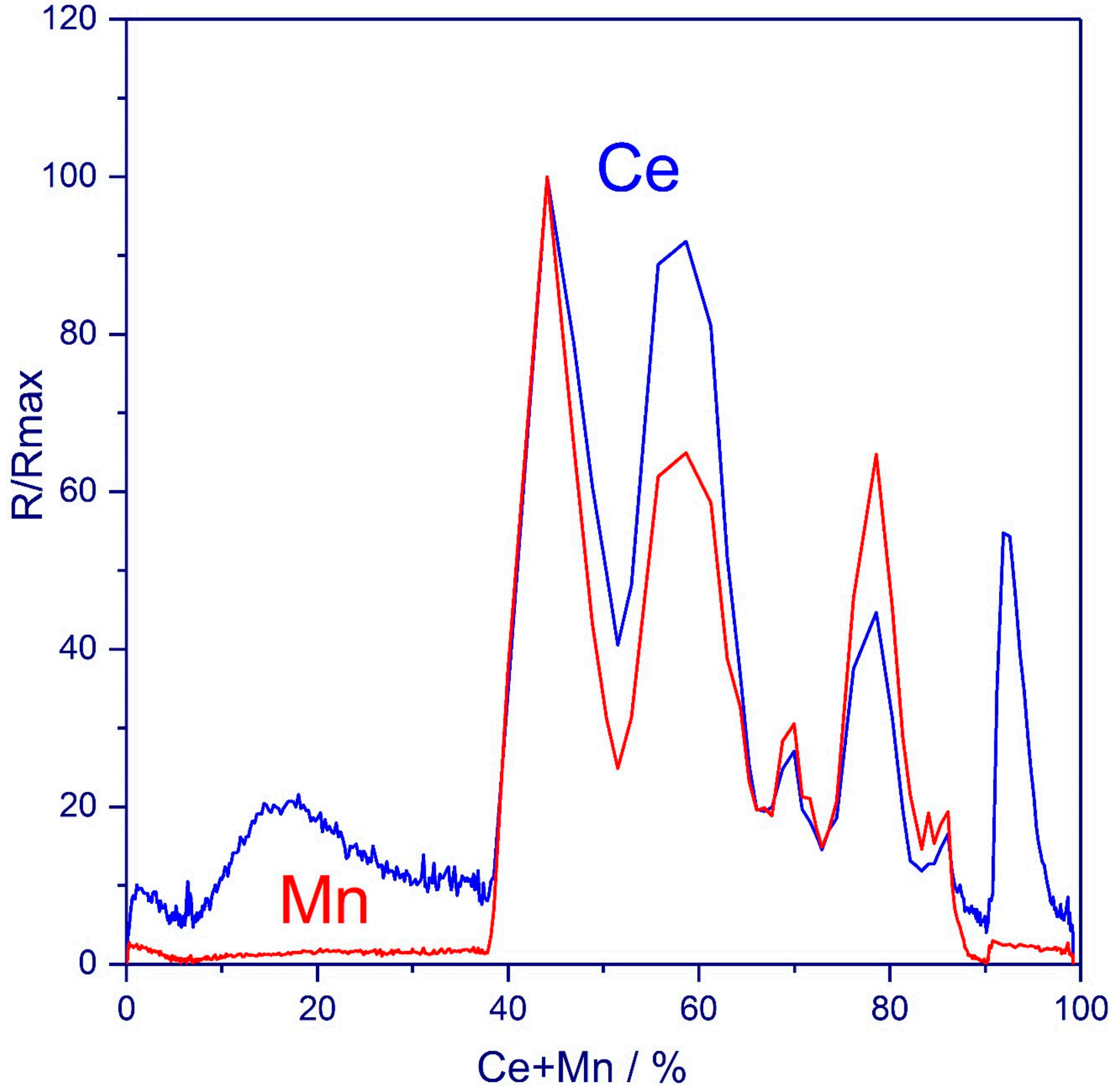

Figure S1. The dissolution curves of Mn and Ce normalized per the maximum dissolution rate (R/Rmax) depending on degree of dissolution Mn and Ce cations for *Mn0.5Ce0.5*.

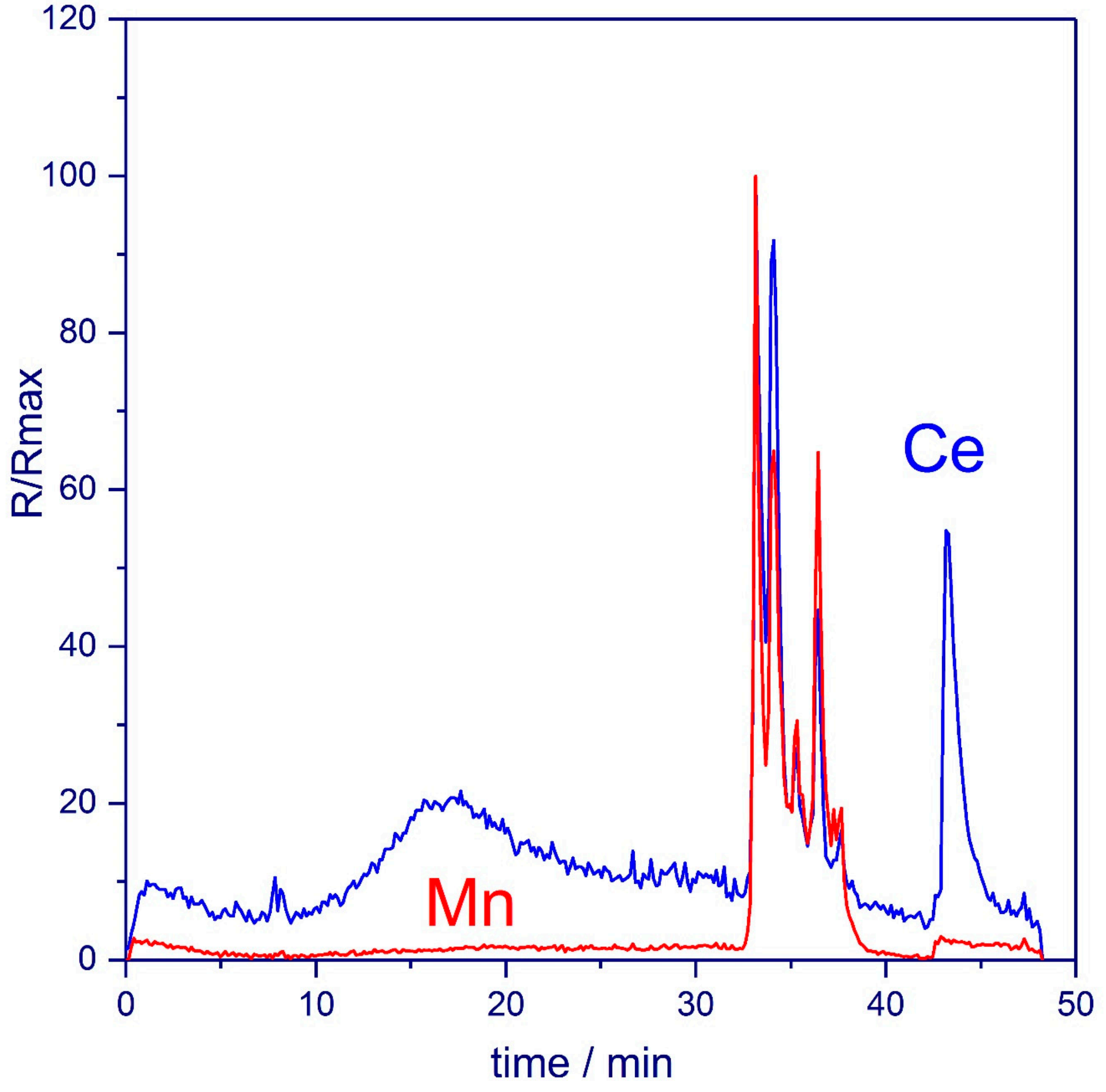

Figure S2. The dissolution curves of Mn and Ce normalized per the maximum dissolution rate (R/Rmax) depending on time for *Mn0.5Ce0.5*.

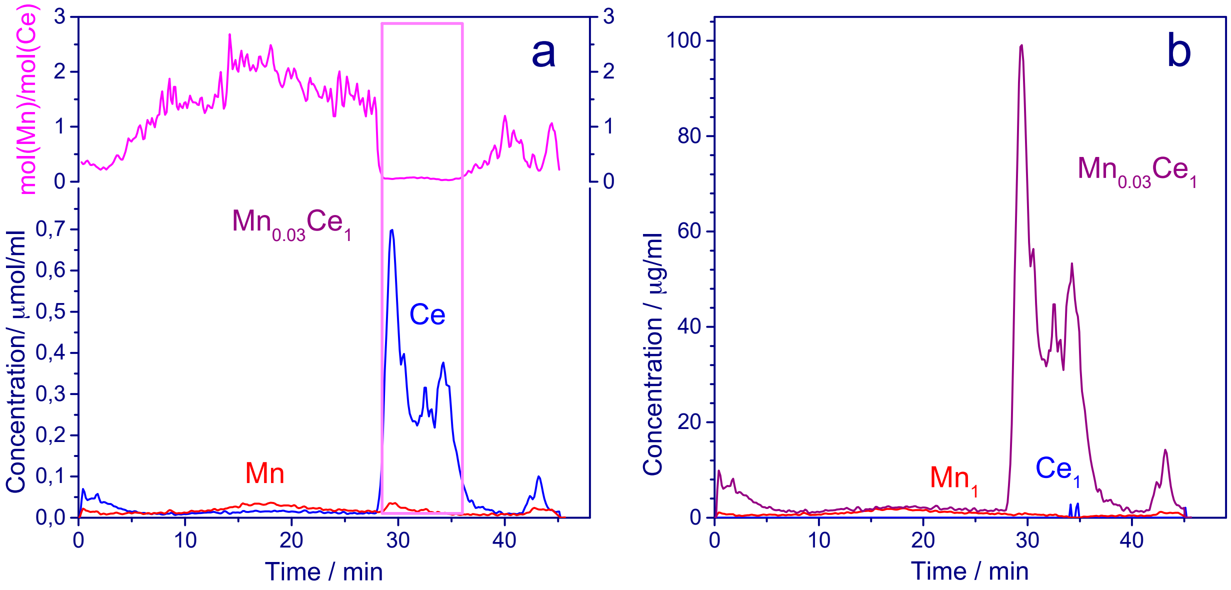

Figure S3. The dissolution curves for manganese and cerium depending on time, the Mn/Ce ratio of the *Mn0.1Ce0.9* catalyst (a). The dissolution curves for Mn_1, Ce_1 and $Mn_{0.03}Ce_1$ phases (b).

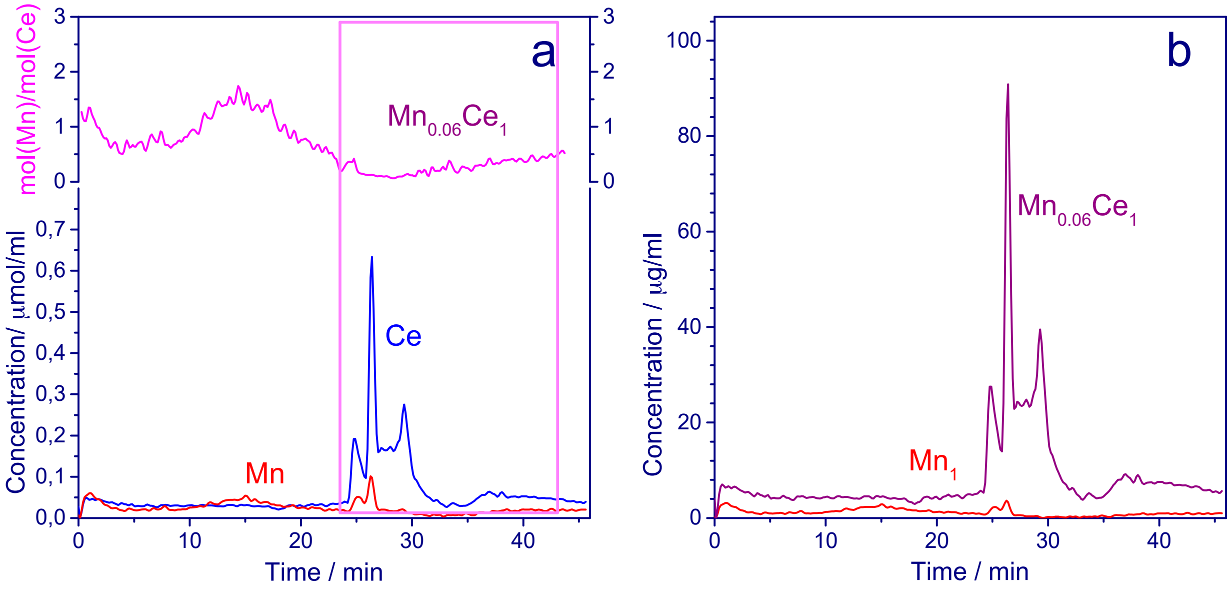

Figure S4. The dissolution curves for manganese and cerium depending on time, the Mn/Ce ratio of the *Mn0.15Ce0.85* catalyst (a). The dissolution curves for Mn_1 and $Mn_{0.06}Ce_1$ phases (b).

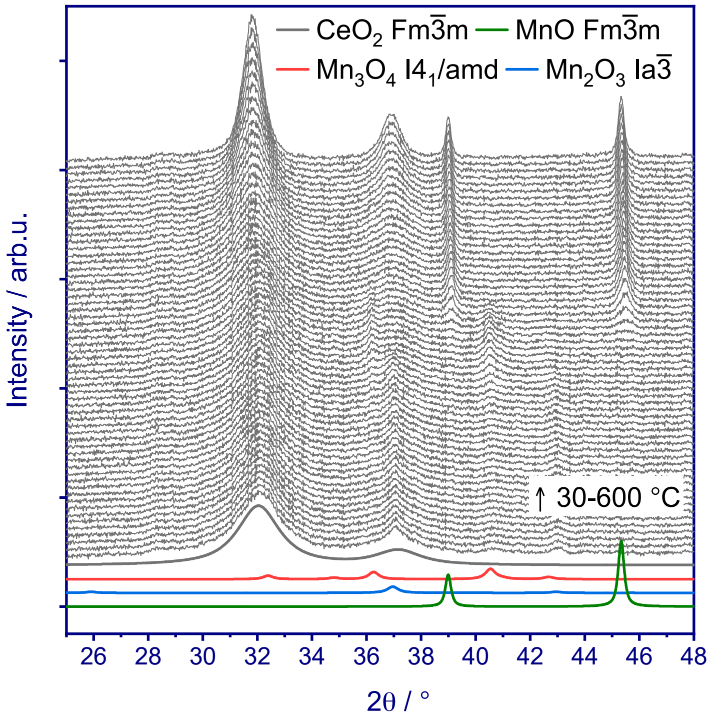

Figure S5. XRD patterns of *Mn0.45Ce0.55* catalyst during *in situ* reduction treatment under hydrogen. Shown below are the model patterns of manganese and cerium oxides. The wavelength of 0.17238 nm.

To further explore the catalytic performance of the *Mn0.45Ce0.55* catalyst, the evolution of CO conversion with time was investigated at 300°C and a contact time of 0.06 s. Fig. S6 shows that the CO conversion does not change significantly and varies in the range of 97.8-99.7%. The initial CO conversion was 98.8% and after 60 hours, it was 98.5%. Thus, the test in time showed the stability of the catalytic properties of *Mn0.45Ce0.55*.

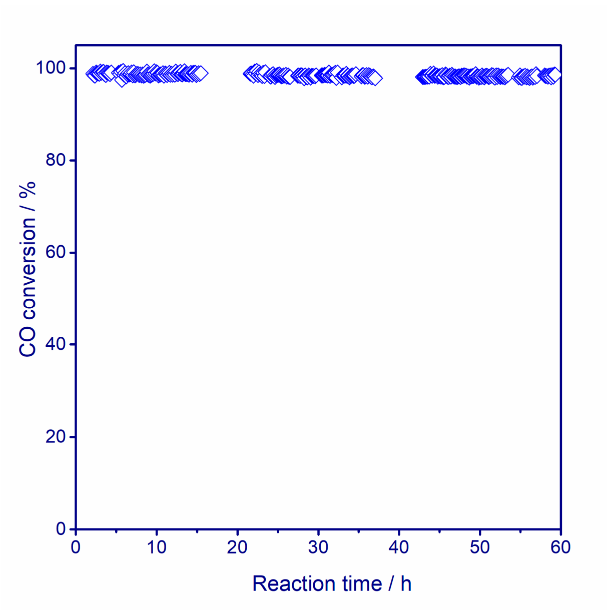

Figure S6. The evolution of CO conversion with time over the *Mn0.45Ce0.55* catalyst.

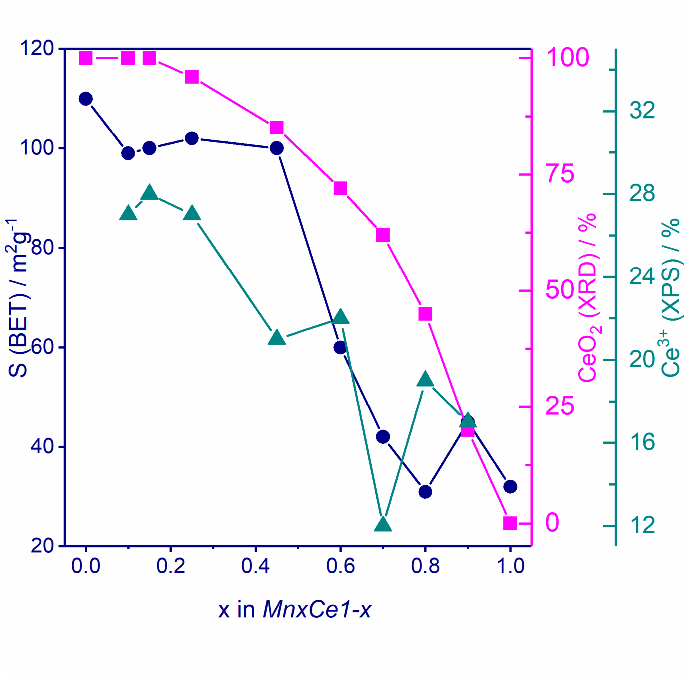

Figure S7. The relation between physiochemical characteristics: the surface area (blue circles), the content of ceria based on XRD calculations in wt% (magenta squares), and the surface content of $Ce^{3+}/Ce^{3+} + Ce^{4+}$ in % based on XPS results (cyan triangles).

1. Malakhov, V.V. and I.G. Vasilyeva, *Stoichiography and chemical methods of phase analysis of multielement multiphase substances and materials.* Russian Chemical Reviews, 2008. **77**(4): p. 351-372.
